# Nephrolithiasis: nutrition as cause or therapeutic tool

**DOI:** 10.1186/1479-5876-11-178

**Published:** 2013-07-26

**Authors:** Irene Brenna, Elena Dogliotti, Annalisa Terranegra, Benedetta Raspini, Laura Soldati

**Affiliations:** 1 Department of Health Sciences, Università degli Studi di Milano, via A. Di Rudini 8, 20142, Milano, Italy; 2 Fondazione Umberto Veronesi, p.zza Velasca 5, 20142, Milano, Italy

## Abstract

Nephrolithiasis is a very common disease with an increasing prevalence among industrialized populations. Kidney stone formation is a complex phenomenon, involving genetic and metabolic patterns, and nutrition can play an important role in this match both as a promoter or as a protective factor. To promote a deeper knowledge of such a challenging disease, clinicians and researchers have met in Rome, Italy, last March 2013, at the International Congress “Nephrolithiasis: a systemic disorder” to discuss patho-physiology and possible treatment of kidney stones. During the meeting, a whole session was dedicated to nutrition, seen both as a cause or a therapeutic tool for nephrolithiasis. Due to its etiopathogenesis, nephrolithiasis is also an ideal model for a nutrigenetics and nutrigenomics approach. Nutrigenomics and nutrigenetic respectively study the effects of a dietary treatment on gene expression and, on the other hand, the impact of an inherited trait on the response to a specific dietary treatment.

## Introduction

Nephrolithiasis is a very common disease in countries with a high socio-economic level as other chronic diseases such as obesity, hypertension, atherosclerosis, cardiovascular disease and type 2 diabetes. In these countries, the prevalence of nephrolithiasis is about 10% [1] and in Italy it is one of the three main causes of hospitalization for nephro-urological reasons, together with renal failure and prostatic hyperplasia [2].

The etiopathogenesis of nephrolithiasis has not yet been fully clarified, although some predisposing factors significantly increase the probability of stones formation such as sex, age, ethnicity, genetics, climate, low water intake, low or high urinary pH, unbalanced diet, overweight or obesity [3]. Starting from epidemiology, in the last thirty years, several large cohort studies have examined the association between diet and stone disease [4,5].

## Discussion

Several studies suggested that the typical industrialized countries diet, which is rich in salt and animal proteins, sugar-sweetened drinks and fructose, leads to high urinary excretion of calcium, uric acid, oxalate and phosphorus, and decreases urinary citrate and pH, thus promoting stone formation [6-8]. On the other hand, a higher consumption of fruit and vegetables seems to be a protective factor for stones, leading to an adequate intake of anti-lithogenic factors such as potassium, magnesium, citrate and phytate and reducing dietary salt intake [4,9,10]. The role of dietary calcium is more controversial. As a matter of fact, a low calcium intake was formerly thought to be protective against the onset of kidney stones, but nowadays it is considered useless, since it promotes the intestinal absorption of oxalate and it has been suggested that it could encourage the onset of bad dietary habits (such as a higher animal protein intake).

An example of nutrigenomics is given by the interaction between two candidate genes for nephrolithiasis, calcium sensing receptor (CaSR) and the tight junction protein claudin 14 with calcium diet intake. One of the most important functions of CaSR is the regulation of renal calcium excretion. The polymorphism R990G of CaSR gene gives a gain of function to CaSR protein [11-13]. This more activated receptor, in comparison to the wild type, leads to hypercalciuria by inhibiting PTH secretion in parathyroid cells as well as by inhibiting calcium transport in thick ascending limb of the loop of Henle (TALH) and collecting duct renal cells. In TALH cells, CaSR modulates the activity of some tight junction proteins. When activated by interstitial calcium, it inhibits claudin 16 translocation to tight junctions to form, together with claudin 19, a paracellular calcium channel. At the same time, it promotes the translocation of claudin 14 that blocks this paracellular calcium channel. The expression of protein claudin 14 was modulated by calcium intake in mice experiments. Under normal dietary condition, equal to 0.61% in calcium, claudin 14 was suppressed by two microRNA molecules, but this effect was reversed with 5% of calcium in the diet [14]. These gene effects, in conjunction with calcium intake levels, may emphasize the predisposition to nephrolithiasis in kidney stone formers.

Beyond the mere association between dietary habits and kidney stones onset, something new about the pathogenesis of nephrolithiasis is emerging, especially about uric acid nephrolithiasis. This particular kind of stones originates from the combination of low urinary volume, hyperuricosuria and low urinary pH, resulting in titration of the soluble urate to the highly insoluble uric acid [15]. Uric acid stone formers, which are often obese, have an increased acid load to the kidney due to both an excessive dietary intake and an increased endogenous acid generation. This is still poorly understood, but seems to involve acquired insulin resistance and the intestinal bacteria [16]. Moreover, these kinds of patients are unable to properly utilize ammonia to buffer urinary protons. This defect seems to be caused by fat infiltration of the kidney, a sort of renal steatosis, due to the high levels of circulating free fatty acids typical of obese patients. This fat infiltration causes tubular lipotoxicity and alters tubular functions. As a result, the kidney uses alternative buffers to carry urinary protons, such as urate, which when titrated precipitates and forms stones. Uric acid nephrolithiasis is therefore a good model to understand how crucial is the interaction between nutrition and metabolic patterns in promoting the development of a complex pathology.

Even though the relationship between dietary habits and nephrolithiasis are being increasingly clear, it could be interesting to study the direct influence of dietary factors on genome, through epigenetic regulation of gene expression. This new kind of approach could lead to a deeper knowledge of nutrition potentiality and to a really personalized diet therapy for kidney stone formers.
